# In mouse model of mixed granulocytic asthma with corticosteroid refractoriness, Bronchom mitigates airway hyperresponsiveness, inflammation and airway remodeling

**DOI:** 10.1186/s10020-024-00888-7

**Published:** 2024-08-11

**Authors:** Acharya Balkrishna, Sandeep Sinha, Anupam Pandey, Surjeet Singh, Monali Joshi, Rani Singh, Anurag Varshney

**Affiliations:** 1 Drug Discovery and Development Division, Patanjali Research Foundation, Haridwar, India; 2 Department of Allied and Applied Sciences, University of Patanjali, Haridwar, India; 3 Patanjali UK Trust, Glasgow, UK; 4 Special Centre for Systems Medicine, Jawaharlal Nehru University, New Delhi, India

**Keywords:** Severe asthma, Mixed granulocytic asthma, Steroid refractoriness, Inflammation, Bronchom, Ayurveda

## Abstract

**Background:**

Asthma is a heterogeneous, inflammatory disease with several phenotypes and endotypes. Severe asthmatics often exhibit mixed granulocytosis with reduced corticosteroid sensitivity. Bronchom is a newly developed Ayurvedic prescription medicine, indicated for the treatment of obstructive airway disorders. The purpose of the present study was to evaluate the in-vivo efficacy of Bronchom in mouse model of mixed granulocytic asthma with steroidal recalcitrance.

**Methods:**

High-performance thin layer chromatography (HPTLC) and Ultra-high performance liquid chromatography (UHPLC) were employed to identify and quantitate the phytometabolites present in Bronchom. The preclinical effectiveness of Bronchom was assessed in house dust mite (HDM) and Complete Freund’s adjuvant (CFA)-induced mixed granulocytic asthma model in mice. High dose of dexamethasone was tested parallelly. Specific-pathogen-free C57BL/6 mice were immunized with HDM and CFA and nineteen days later, they were intranasally challenged with HDM for four consecutive days. Then the mice were challenged with nebulized methacholine to evaluate airway hyperresponsiveness (AHR). Inflammatory cell influx was enumerated in the bronchoalveolar lavage fluid (BALF) followed by lung histology. Additionally, the concentrations of Th2 and pro-inflammatory cytokines was assessed in the BALF by multiplexed immune assay. The mRNA expression of pro-inflammatory cytokines and Mucin 5AC (MUC5AC) was also evaluated in the lung.

**Results:**

HPTLC fingerprinting and UHPLC quantification of Bronchom revealed the presence of bioactive phytometabolites, namely, rosmarinic acid, gallic acid, methyl gallate, piperine, eugenol and glycyrrhizin. Bronchom effectively reduced AHR driven by HDM-CFA and the influx of total leukocytes, eosinophils and neutrophils in the BALF. In addition, Bronchom inhibited the infiltration of inflammatory cells in the lung as well as goblet cell metaplasia. Further, it also suppressed the elevated levels of Th2 cytokines and pro-inflammatory cytokines in the BALF. Similarly, Bronchom also regulated the mRNA expression of pro-inflammatory cytokines as well as MUC5AC in mice lungs. Reduced effectiveness of a high dose of the steroid, dexamethasone was observed in the model.

**Conclusions:**

We have demonstrated for the first time the robust pharmacological effects of an herbo-mineral medicine in an animal model of mixed granulocytic asthma induced by HDM and CFA. The outcomes suggest the potential utility of Bronchom in severe asthmatics with a mixed granulocytic phenotype.

## Background

Bronchial asthma is widely recognized as a heterogeneous obstructive airway disease with varying underlying disease processes. Accordingly, several clinical phenotypes of asthma have been recognized, including allergic asthma, non-allergic asthma, adult onset asthma, asthma with persistent airflow limitation and asthma with obesity (GINA 2023). In addition to these established phenotypes, considerable variation also exists in asthmatic individuals with respect to the proportion of granulocytes detected in their sputum and accordingly asthma can also be classified as eosinophilic, neutrophilic, mixed granulocytic and paucigranulocytic (Xiao et al. 2022). Of these subtypes, in mixed granulocytic asthma, combined elevations in both eosinophils and neutrophils are observed in sputum samples. When compared to patients exhibiting eosinophilia and neutrophilia alone, the patients demonstrating mixed granulocytosis have been reported to have the lowest pulmonary function and consequent higher healthcare costs. These patients also exhibit a greater decline in lung function and are at a higher risk of developing asthma exacerbations with time (Hastie et al. 2020). Accordingly, mixed granulocytic asthma can be recognized as one of the most severe phenotype, which remains uncontrolled despite the use of corticosteroid therapies. Such patients may require systemic along with inhaled corticosteroids to achieve some degree of asthma control. They are also candidates for molecular-targeted therapies involving use of monoclonal antibodies against multiple inflammatory mediators involved in the disease pathogenesis (Chung et al. 2014). However, the pharmacoeconomic constraints and worldwide access to biologics continue to remain as unaddressed concerns, along with lack of efficacy and treatment limiting adverse effects (Caminati et al. 2021; Faverio et al. 2023). Hence there exists an unmet medical need for new efficacious, safe, affordable, patient compliant and readily available medicines for patients suffering from mixed granulocytic severe asthma.

Bronchom is a novel herbo-mineral Ayurvedic prescription medicine, which has been additionally enriched with minerals containing elemental calcium. It has been particularly formulated for the treatment of obstructive airway diseases, as per the ancient medicinal texts. We have recently reported the preclinical effectiveness of Bronchom in murine model of allergic asthma induced by HDM (Balkrishna et al. 2024). Bronchom is predominantly based on the following herbs, *Glycyrrhiza glabra*,* Solanum xanthocarpum*,* Pistacia integerrima*,* Zingiber officinale*,* Piper longum* etc. The detailed phytoconstituents of Bronchom have been previously described (Balkrishna et al. 2024). These specific phyto- and mineral constituents of Bronchom have been traditionally used in Ayurveda and other traditional medicinal systems for the treatment of obstructive lung diseases, for centuries.

The objective of the present study was to assess the efficacy of Bronchom in a mouse model of mixed granulocytic asthma, which has been reported to develop subsequent to immunization of mice with house dust mite (HDM) and complete Freund’s adjuvant (CFA) followed by intranasal HDM challenge (Alba et al. 2015; Menson et al. 2020). In the present study, Bronchom was administered orally as a gavage, two weeks prior to initiation of the disease induction process. Subsequently, mice were immunized subcutaneously with an emulsion of HDM and CFA, followed by repeated intranasal HDM challenge for four consecutive days, starting from the nineteenth day post-sensitization. Endpoint parameters evaluated at study termination included, airway hyperresponsiveness (AHR) to nebulized methacholine, cytological analysis of the bronchoalveolar lavage fluid (BALF), estimation of Th2 and proinflammatory cytokines in the BALF, histological analysis of the lung and gene expression analysis in the lung. Additionally, detailed phytochemical analysis of Bronchom was also conducted to identify and quantify potential biologically active marker phytometabolites in the medicine, with an intent to elucidate the findings of the in-vivo experiments.

## Materials and methods

### Test article, chemicals and reagents

Bronchom (Internal batch number: CHIH/BROA/0921/1681) was sourced from Divya Pharmacy, Haridwar, India. The phytoconstituents of Bronchom have been elaborated in our previously published research article (Balkrishna et al. 2024). Complete Freund’s adjuvant (CFA) supplemented with 4 mg/mL of heat inactivated *Mycobacterium tuberculosis*, (Strain H37Ra), was procured from Chondrex, USA (Cat. # 7001). House dust mite (HDM), consisting of the whole bodies of *Dermatophagoides pteronyssinus* (Part # XPB70D3A2.5) was purchased from Stallergenes Greer, USA. Dexamethasone (Product # D4902), rosmarinic acid (Product # R4033, Purity, 98%), gallic acid (Product # 91215, Purity, 97%), piperine (Product # P49007, Purity, 97%), eugenol (Product # 35995, Purity, 99%) and Giemsa stain (Product # 48900) were bought from Sigma Aldrich, USA. Succinylcholine (Product # S0149), methacholine (Product # M0073), methyl gallate (Product # G0017, Purity, 99%) and glycyrrhizin (Product # G008, Purity, 93%) were procured from TCI Chemicals (India) Pvt. Ltd, India. The concentrations of Th2 and pro-inflammatory cytokines in the bronchoalveolar lavage fluid were evaluated by employing customized multiplexing kit, purchased from Merck, USA. TRIzol reagent (Cat. # 15596018), Verso cDNA synthesis kit (Cat. # AB-1453/B) and PowerUp SYBR green master mix (Cat. # A25742) were purchased from Thermo Fisher Scientific, USA. The powder for preparing Hank’s balanced salt solution (HBSS) was bought from Himedia Laboratories, India (Cat. # TS1098). Methylcellulose-4000 cps (Article # 04635) was procured from Loba Chemie Pvt. Ltd., India. Standard pellet diet (5L79-18% protein, manufactured by Purina Lab diet, USA) was purchased from Hylasco Biotechnology (India) Pvt. Ltd., India. Animal bedding material, Sparcobb™ was purchased from Sparconn Life Sciences, India.

**Table 1:** Phytocompounds identified and quantified in Bronchom by HPTLC analysis, as depicted in Fig. 2

S. no.	Phytocompound detected	Mobile phase	Retention factor	Content in Bronchom (µg/mg)
1	Rosmarinic acid	A	0.27	0.54
2	Gallic acid	A	0.34	4.12
3	Methyl gallate	A	0.46	3.47
4	Glycyrrhizin	B	0.46	17.50
5	Piperine	A	0.53	4.27
6	Eugenol	A	0.74	1.73

Mobile Phase A: Ethyl acetate, toluene and formic acid (9: 10: 1 v/v/v)

Mobile Phase B: Ethyl acetate, formic acid, acetic acid and water (10: 1.1: 1.1: 2.3 v/v/v/v)

### High-performance thin liquid chromatography (HPTLC) analysis of Bronchom

Standard compounds were weighed and dissolved in methanol to yield a stock solution of concentration, 1000 µg/mL. Subsequently working stock solutions of concentrations 100 µg/mL were prepared for methyl gallate (MG), gallic acid (GA), eugenol (EU), piperine (PI) and glycyrrhizin (GY), whereas for rosmarinic acid (RA), a working stock solution of 10 µg/mLwas made, by using methanol as the diluent. Further, the solution of Bronchom was prepared by adding 10 mL of a solvent comprised of methanol: water (80:20 v/v) to 500 mg of Bronchom powder. This was followed by sonication for 20 min and centrifugation at 6000 rpm for 10 min. The resultant clear solution was then collected and used for analysis. For conducting the analysis of Bronchom, an HPTLC system, equipped with an automatic TLC sampler (ATS4), TLC scanner 4, TLC Visualizer and integrated software winCATS (Version 1.4.10) was used (CAMAG, Switzerland). HPTLC was performed on a pre-coated silica gel 60 _F254_ (Cat. # 1.05554.0007), aluminium backed thin layer chromatography (TLC) plate (Merck, Germany). For fingerprint analysis, 15 µL of each standard and 10 µL of test sample were applied as a 8 mm band using spray-on technique on the TLC plate. With an objective of identifying and quantifying methyl gallate, rosmarinic acid, gallic acid, piperine and eugenol in Bronchom powder, the plate was developed using CAMAG twin trough chamber pre-saturated for 20 min with mobile phase A (containing ethyl acetate, toluene and formic acid in the ratio of 9: 10: 1). For the identification and quantification of glycyrrhizin, mobile phase B was utilized, which comprised of ethyl acetate, formic acid, acetic acid and water in the ratio of 10: 1.1: 1.1: 2.3. The TLC plate was developed up to 70 mm, following which, the developed plate was dried under warm air and subsequently visualized under ultraviolet light (wavelength, 254 nm). The TLC image was additionally scanned for obtaining 3D chromatograms at 280 nm and 254 nm.

For quantification, different concentrations of each standard was applied and on the basis of the obtained linearity plot, concentrations of specific phytometabolites was quantified, under same chromatographic conditions. On the basis of the respective maximum absorbance, scanning was conducted at 254 nm for glycyrrhizin, 280 nm for gallic acid, methyl gallate and eugenol and 430 nm for piperine and rosmarinic acid, with Slit dimension of 6 × 0.45 mm, micro scanning speed at 20 mm/s, and with data resolution of 100 μm/step. Deuterium lamp was used under absorption mode.

### Experimental animals and husbandry conditions

Female, specific pathogen free (SPF) C57BL/6 mice, 6–8 weeks of age and weighing between 20 and 25 g were purchased from Hylasco Biotechnology (India) Pvt. Ltd., India, a Charles River Laboratories (USA)-licenced experimental animal supplier. All the experimental procedures conducted in this study conformed with the guidelines specified by Committee for Control and Supervision of Experiments on Animals (CCSEA), Department of Animal Husbandry and Dairying, Ministry of Fisheries, Animal Husbandry and Dairying, Government of India. Before initiating the experiments, the experimental protocol was comprehensively reviewed and consecutively approved by the Institutional Animal Ethics Committee of Patanjali Research Foundation, vide approval number PRIAS/LAF/IAEC-104. The laboratory animal facility, where the study was conducted is also duly registered with CCSEA vide registration number 1964/PO/Rc/S/17/CPCSEA.

After their arrival at the facility, the mice were transferred to a quarantine room where they were maintained for one-week and observed for any health-related abnormalities by a staff veterinarian. Seven days later, upon confirmation of their satisfactory health status, mandatorily required for the conduct of the study, animals were transferred to an experimental room for the study, where mice were housed in polypropylene cages of standard dimensions. The temperature of the experimental room throughout the course of the study ranged from 21 to 25 °C and the relative humidity was maintained between 50 and 60%, along with a 12-hour light/dark cycle. Animals were offered an unrestricted supply of gamma-irradiated (25 kGy), pelleted laboratory animal diet. Similarly, reverse osmosis (Make: Merck, India) water was also provided *ad libitum* in autoclaved polypropylene bottles.

### Calculation of the doses for the in-vivo study

Doses of Bronchom selected for the study were calculated on the basis of the differences in the body surface areas of mice and humans. The clinically prescribed dose of Bronchom is 2000 mg/day, in two distributed doses of 1000 mg, respectively in a day. Consequently, the therapeutic dose for a 60 kg-human would be 2000/60 i.e. 33.33 mg/kg/day. The mouse equivalent dose (in mg/kg) was arrived at, by multiplying the human equivalent dose (in mg/kg) by factor of 12.3 (Nair and Jacob 2016). Accordingly, the equivalent dose for mice was computed to be 409.99 mg/kg/day. Rounding off to the nearest hundreds, 400 mg/kg/day or 200 mg/kg, b.i.d. was considered to be the mouse equivalent of the prescribed human dose. Further, with an intention of arriving at a probable dose-response relationship in the studied experimental readouts, the remaining doses chosen were from 1/3^rd^ to 6 times the recommended therapeutic dose i.e. 60, 600 and 1200 mg/kg, b.i.d. respectively.

In a recently published in-vivo study conducted by our group, oral administration of dexamethasone at the dose of 1 mg/kg/day significantly attenuated airway hyperresponsiveness and the causative airway remodelling changes in mouse model of house dust mite-induced allergic airway inflammation (Balkrishna et al. 2024). However, in the present study, we chose to employ dexamethasone at a 2.5-fold higher dose (2.5 mg/kg/day), due to the previously reported subdued efficacy of the selected dose in mixed granulocytic asthma model induced by HDM and CFA, subsequent to intraperitoneal administration (Menson et al. 2020).

### Compound administration and generation of the mouse model of mixed granulocytic airway inflammation

After conclusion of the quarantine period, the animals were weighed followed by randomization, based on their respective body weights. Thereafter, mice were assigned to seven groups consisting of six animals in each group, as described below:

Group 1: Normal control (NC) group treated with 0.5% methylcellulose, *bis in die* (b.i.d. i.e. twice daily).

Group 2: Disease control (DC) group treated with 0.5% methycellulose, b.i.d.

Group 3: Treated with Dexamethasone-2.5 mg/kg (DEX-2.5), *quaque die* (q.d. i.e. once daily).

Group 4: Treated with Bronchom-60 mg/kg (BC-60), b.i.d.

Group 5: Treated with Bronchom-200 mg/kg (BC-200), b.i.d.

Group 6: Treated with Bronchom-600 mg/kg (BC-600), b.i.d.

Group 7: Treated with Bronchom-1200 mg/kg (BC-1200), b.i.d.

**Fig. 1:**
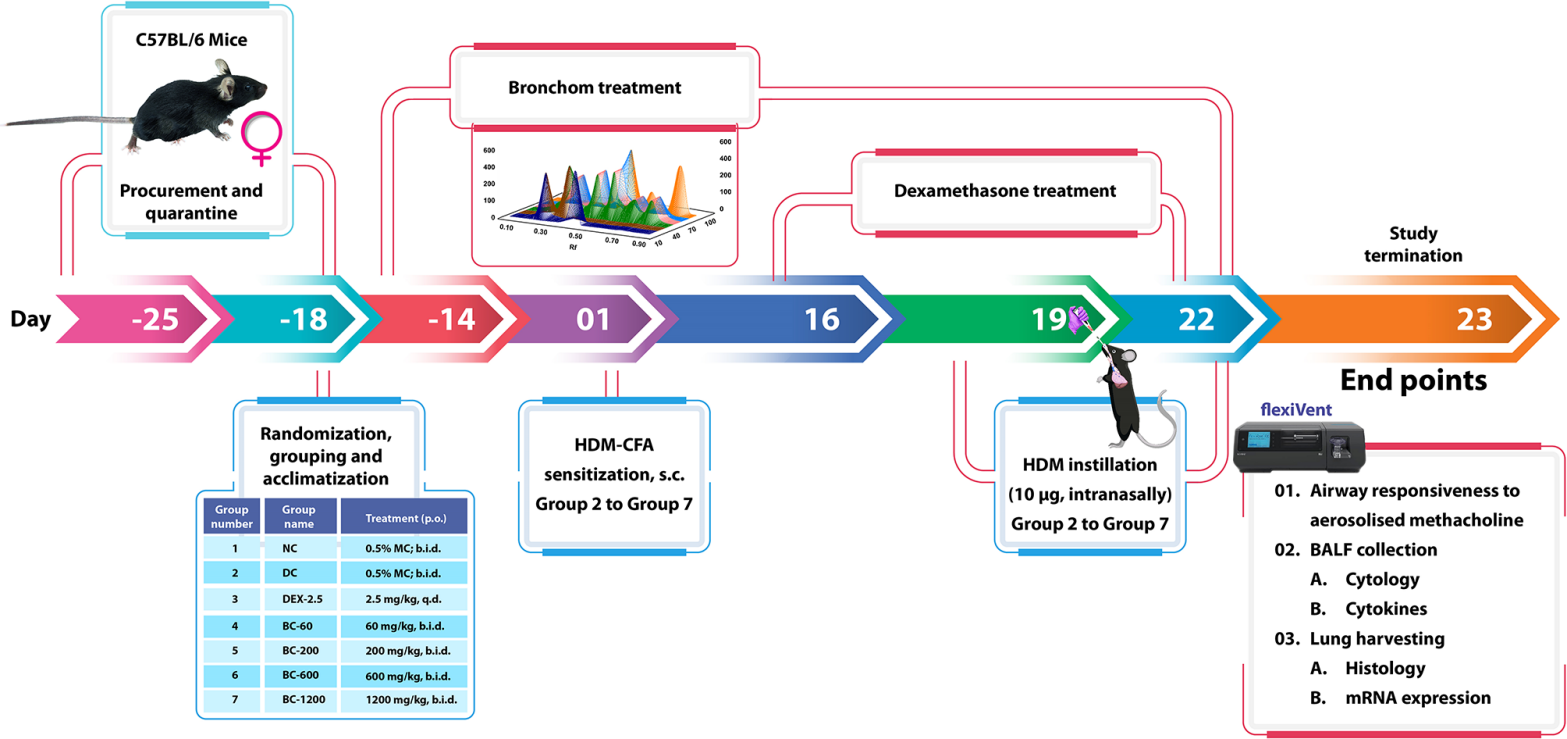
Schematic representation of the in-vivo experiment. After completion of quarantine and acclimatization, animals were administered the test compounds, prophylactically for 14 days. During this experimental period, mice allocated to normal-control (NC), disease-control (DC) and dexamethasone (DEX)-treated group were administered 0.5% methylcellulose (MC) orally, twice daily. The animals that received Bronchom (BC) were administered the formulation as a suspension in 0.5% MC by gavage twice daily, in escalating doses of 60, 200 600 and 1200 mg/kg, b.i.d. Thereafter, the mice of all the groups except the normal-control group received 100 µL of an emulsion containing house dust mite (HDM) extract and complete Freund’s adjuvant (CFA) as elaborated in the Materials and Methods section by subcutaneous (s.c.) route. On days 19, 20, 21 and 22 all the animals except those which were allocated to the normal-control group were intranasally instilled with 10 µg of house dust mite extract (dissolved in 20 µL of saline). The dexamethasone treated group received the compound from day 16 till the end of the experiment at the dose of 2.5 mg/kg, once daily by oral route. On day 23, twenty-four hours after the last HDM instillation, mice were anaesthetized and airway responsiveness to nebulized methacholine was measured. Subsequently, the animals were humanely sacrificed and bronchoalveolar lavage fluid (BALF) was collected for enumeration of total leukocytes, eosinophils and neutrophils and for the estimation of the concentration of cytokines. Thereafter, both the lungs of the mice were harvested for histological assessments in the left lung and for mRNA expression studies of pro-inflammatory cytokines and MUC5AC in the right lung.

The allocation of mice is elaborated in Fig. 1 as well. After completion of randomization, the animals were allowed to acclimate to the experimental room for four days.

Post-acclimatization, the mice received Bronchom orally by using a gavage needle, fourteen-days prior immunization of the animals with HDM and CFA. 0.5% methylcellulose (MC) solution was employed as the suspending agent to prepare a suspension of the test compounds and the animals received the compounds in a dose volume of 10 mL/kg.

In order to create the murine model of mixed granulocytic asthma, the animals allocated to Group 2 to Group 7, were anaesthetized with isoflurane on Day 1 and were subcutaneously administered 100 µL of an emulsion of HDM and CFA containing 50 µL of Complete Freund’s Adjuvant (supplemented with 4 mg/mL of heat inactivated *Mycobacterium tuberculosis*, strain H37Ra) and 25 µg of HDM extract (based on the protein content), dissolved in 50 µL saline. Based on the volume of CFA injected, the dose of *Mycobacterium tuberculosis* received by animals was 200 µg. These doses of CFA and HDM, employed in the current experiment were selected on the basis of a previous study (Menson et al. 2020). Animals allocated to Group 1 were administered saline by subcutaneous route. Dexamethasone administration was initiated on day 16. On days 19, 20, 21 and 22, the animals assigned to Group 2 to Group 7 were challenged with 10 µg of HDM extract dissolved in 20 µL of saline by intranasal instillation, under transient isoflurane anaesthesia, whereas Group 1 animals received 20 µL of normal saline, intranasally.

### Measurement of airway responsesiveness to nebulized methacholine

On day 23, twenty-four hours after the final HDM intranasal instillation, the animals were anaesthetized with intraperitoneally administered thiopentone sodium (50 mg/kg), and tracheostomy was performed. This was followed by insertion of an 18-gauge blunted needle in the trachea, which was then connected to a flexiVent™ instrument (Emka-Scireq, Canada) for the measurement of airway hyperresponsiveness. The mice were ventilated at 150 breaths/min with a tidal volume of 0.25 mL. A positive end-expiratory pressure of 3 cm H_2_O was maintained throughout the experiment. Before pulmonary function testing, mice were subjected to respiratory paralysis with an intraperitoneal injection of succinylcholine chloride (7.5 mg/kg). Thereafter, baseline measurements of respiratory mechanics were assessed prior to the challenge with increasing concentration of aerosolized methacholine (1.56-50 mg/mL). The respiratory system resistance (Rrs) was determined after challenge with each of the concentration of methacholine. Furthermore, the Rrs obtained after challenging the animals allocated to Group 2 to Group 7, at the concentration of 50 mg/mL of methacholine was additionally normalized with the response obtained in the Normal-control group at 50 mg/mL. The percentage protection from AHR for Group 3 to Group 7 was calculated by using the below mentioned formula:

$$\begin{aligned}&\%\:Protection\:from\:\:AHR\:\\&\quad =\frac{\begin{array}{c} (Normalized\:Rrs\:in \\ Disease\:control\:group) \end{array}- \begin{array}{c}(Normalized\:Rrs\:in \\ Treated\:groups)\end{array}}{Normalized\:Rrs\:in\:Disease\:control\:group}\:\times\:100\end{aligned}$$

### Collection of bronchoalveolar lavage fluid and determination of its cellularity

After the measurement of AHR, the animals were humanely sacrificed with an intraperitoneal injection of thiopentone (150 mg/kg). BALF samples were retrieved through the tracheal cannula as described previously (Balkrishna et al. 2024), by using HBSS to sustain the cell viability.The BALF supernatants were stored at -80 °C for the estimating the concentrations of cytokines, whereas the cell pellets were resuspended in HBSS for enumerating the total and differential leukocyte counts. Total leukocytes were determined by using a hematology analyzer (BC5000 Vet, Mindray, China). Further, the differential leukocyte counts were enumerated in smears of the cell suspension, obtained by using a cytocentrifuge (Medspin5; Medilabsolutions, India). The cyto-smears were subjected to staining with Giemsa stain. Thereafter, three hundred leukocytes were manually counted by employing a brightfield microscope (Olympus BX43, Perkin Elmer, USA) and the following cells were identified by an observer blinded to the treatment, based on their morphology: macrophages, eosinophils, lymphocytes, neutrophils and basophils. The enumerated cells were expressed as a percentage and the absolute counts of each of the identified leukocytes were calculated by using the formula:

$$\begin{aligned}\:Absolute\:cell\:count=&\\&\%\:of\:the\:identified\:leukocytes\\&\:\times\:Total\:cell\:count\end{aligned}$$

### Collection of lungs and processing for histological evaluation

Lungs were harvested from the animals subsequent to the collection of BALF. The left lung was transferred to 10% neutral buffered formalin and the right lung was flash frozen in liquid nitrogen and then instantly stored at -80 °C for mRNA expression experiments. The left lung was processed by utilizing standard procedures for histological evaluation. Thereafter, 3–5 μm sections were subjected to staining with hematoxylin and eosin (H&E) stain to detect the influx of inflammatory cells in the lung and Periodic Acid-Schiff (PAS) stain for determination of goblet cell metaplasia, as reported previously (Balkrishna et al. 2020). The histological evaluation was conducted by a trained veterinary histopathologist who was blinded to the treatment.

### Measurement of cytokines and chemokines in the BALF

Th2 (IL-4 and IL-5) and pro-inflammatory cytokines (TNF-α, IL-6 and IL-1β) were quantified in the BALF obtained from five animals per experimental group by a multiplexing assay that utilized a customized MILLIPLEX^®^ Mouse Cytokine Magnetic Bead Panel (Merck, USA). The employed panel, based on Luminex^®^ xMAP^®^ technology, is a customized multiplexing kit, used for simultaneous quantification of the selected murine cytokines in various ex-vivo matrices, including BALF. The cytokine concentrations were estimated in 25 µL of undiluted BALF samples on a MAGPIX^®^ analyzer (Merck, Germany), according to the manufacturer’s instructions. Experiments were conducted at Merck High-End Skill Development Centre, Council of Scientific and Industrial Research-Institute of Microbial Technology, Chandigarh, India in a blinded manner.

### Quantitative real-time polymerase chain reaction (qRT-PCR) for mRNA expression in lung tissue

mRNA expression of IL-6, IL-1β and MUC5AC genes were performed in the lung tissues obtained from four animals of each of the study groups. Total RNA was isolated from lung tissue using TRIzol reagent. Subsequently, 1 µg of RNA was reverse transcribed using Verso cDNA synthesis kit according to the manufacturer’s instructions. qRT-PCR was performed using at least 10 ng cDNA (2 µL) in a 10 µL reaction containing 0.5 pM of each primer, 5 µL PowerUp SYBR green master mix, and 2 µL of milli-Q water. qRT-PCR analysis was conducted on the qTOWER3 G instrument (Analytik-Jena, Germany) with following cycling condition: 95 °C for 5 min, followed by 40 cycles of denaturation at 95 °C for 30 s, annealing at 60 °C for 30 s, extension at 72 °C for 30 s and final extension at 72 °C for 5 min. Peptidylprolyl Isomerase A (PPIA) was used as a housekeeping gene for normalizing the data. mRNA expression levels were calculated using 2^(-ΔΔCt) method (Livak and Schmittgen 2001). The sequence of forward and reverse primers are as follows:

PPIA Forward: 5′-GGTGGTGACTTTACACGCCA-3′.

PPIA Reverse: 5′-TCTCCGTAGATGGACCTGCC-3′.

IL-6 Forward: 5′-TGGAGTCACAGAAGGAGTGGCTAAG-3′.

IL-6 Reverse: 5′-TCTGACCACAGTGAGGAATGTCCAC-3′.

IL-1β Forward: 5′-CCCTGCAGCTGGAGAGTGTGGA-3′.

IL-1β Reverse: 5′-TGTCTCTGCTTGTGAGGTGCTG-3′.

MUC5AC Forward: 5′-GTGGTTTGACACTGACTTCCC-3′.

MUC5AC Reverse: 5′-CTCCTCTCGGTGACAGAGTCT-3′.

### Statistical analysis

All the data for the studied parameters were compiled from each of the study groups and expressed as mean ± standard error of mean (SEM). Statistical analysis was performed using GraphPad Prism version 10.1.1 software (GraphPad Software, USA). For the AHR data representing increase in Rrs at each concentration of MCh, a two-way analysis of variance (ANOVA) was employed followed by Tukey’s post-hoc multiple comparison test to calculate the statistical differences between the mean values. For the remaining end point parameters, one-way ANOVA followed by Dunnett’s multiple comparison post-hoc test was used. A p value < 0.05 was considered to be statistically significant.

## Results

### HPTLC fingerprinting of Bronchom reveals the presence of phytometabolites known to possess disease-modifying effects in airway disorders

**Fig. 2:**
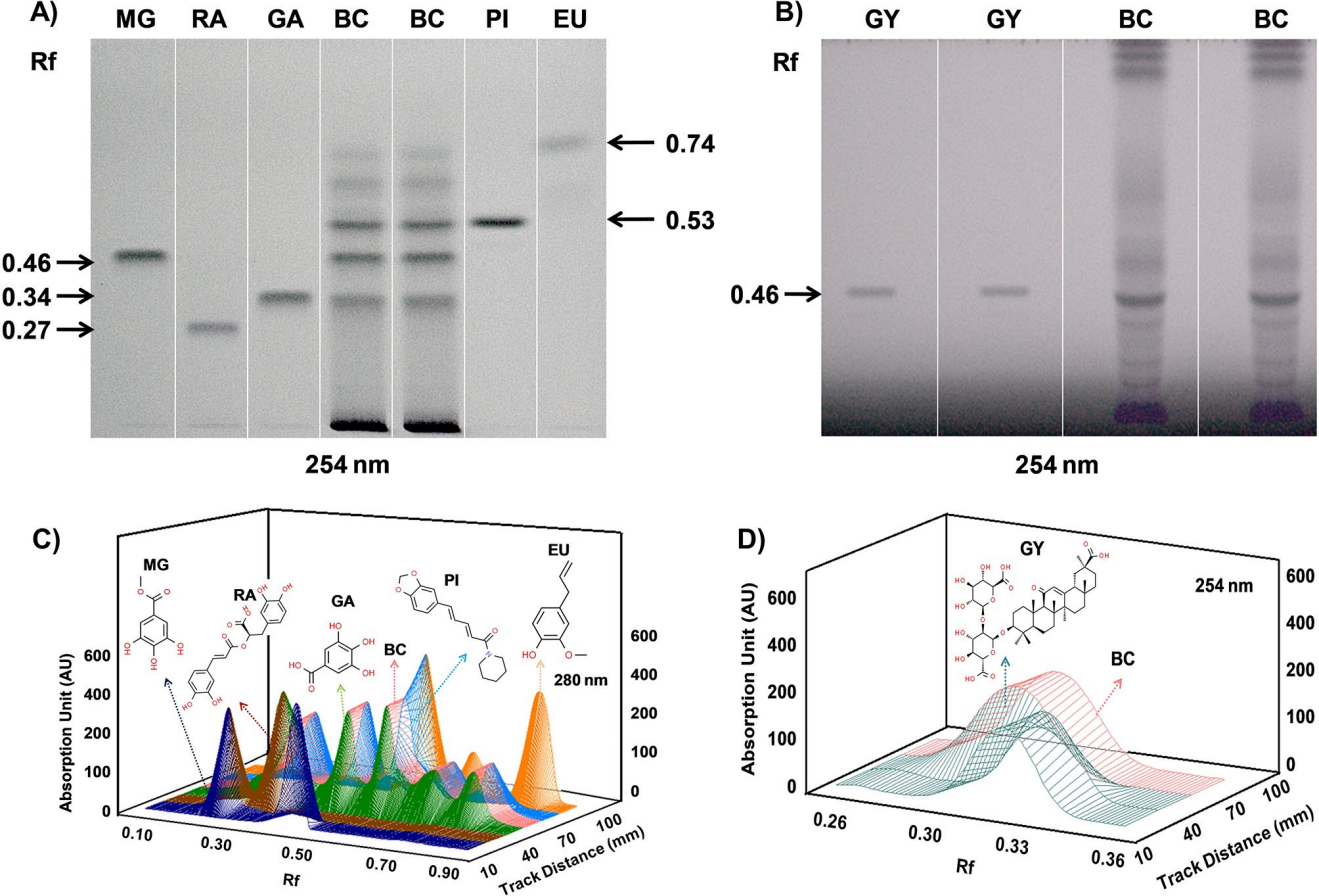
High-performance thin layer chromatography (HPTLC) of Bronchom. Phytocompounds present in Bronchom were analyzed by HPTLC fingerprinting and compared to pure reference standards. Six compounds were identified and subsequently quantified in Bronchom as depicted in Table 1. (**A**) HPTLC fingerprint of Bronchom at 254 nm showing the presence of marker compounds methyl gallate (MG, Rf: 0.46), rosmarinic acid (RA, Rf: 0.27), gallic acid (GA, Rf: 0.34), piperine (PI, Rf: 0.53) and eugenol (EU, Rf: 0.74). (**B**) HPTLC 3-D chromatogram of Bronchom at 280 nm, with respect to reference standards methyl gallate (MG), rosmarinic acid (RA), gallic acid (GA), piperine (PE) and eugenol. (**C**) HPTLC fingerprint of Bronchom at 254 nm demonstrating the presence of marker compound, glycyrrhizin (GY, Rf: 0.46). (**D**) HPTLC 3-D chromatogram of Bronchom at 254 nm, with respect to reference standard, glycyrrhizin (GY). The chemical structures of the identified phytochemicals have been incorporated along with the chromatograms, and have been obtained from www.chemspider.com

The phytometabolites in Bronchom were determined and quantified by employing the HPTLC platform. Bronchom solution underwent separation on TLC plates, which were then exposed at 254 nm to identify the phytochemicals, by comparing with the obtained retention factor (Rf) values of the known standard compounds. HPTLC analysis revealed that mobile phase A enabled the separation of rosmarinic acid (RA), gallic acid (GA), methyl gallate (MG), piperine (PI) and eugenol (EU) with Rf values of 0.27, 0.34, 0.46, 0.53 and 0.74 respectively (Fig. 2A), whereas, mobile phase B facilitated the separation of glycyrrhizin (GY) with an Rf value of 0.46 (Fig. 2B). The identified phytocompounds were further subjected to quantification at distinct wavelengths. The resultant contents of the identified phytocompounds present in each milligram of Bronchom is depicted in Table 1. In addition, 3-D chromatograms were generated at 280 nm to corroborate the presence of RA, GA, MG, PI and EU and at 254 nm to validate the presence of GY (Fig. 2C and D) in Bronchom.

In the previous study, detailed phytochemical analysis of Bronchom has been performed by employing ultra-high performance liquid chromatography (UHPLC) technique. Phytometabolites identified using UHPLC revealed that each milligram of Bronchom contained 8.281 µg of glycyrrhizin, 2.411 µg of piperine, 0.308 µg of 6-gingerol, 3.407 µg of gallic acid, 0.092 µg of protocatechuic acid, 1.393 µg of methyl gallate, 0.793 µg of rosmarinic acid and 2.332 µg of eugenol. The identification and quantification of these phytometabolites has been depicted in our previously published study (Balkrishna et al. 2024).

### HDM-CFA driven airway hyperresponsiveness in HDM-CFA sensitized mice is effectively suppressed by Bronchom

**Fig. 3:**
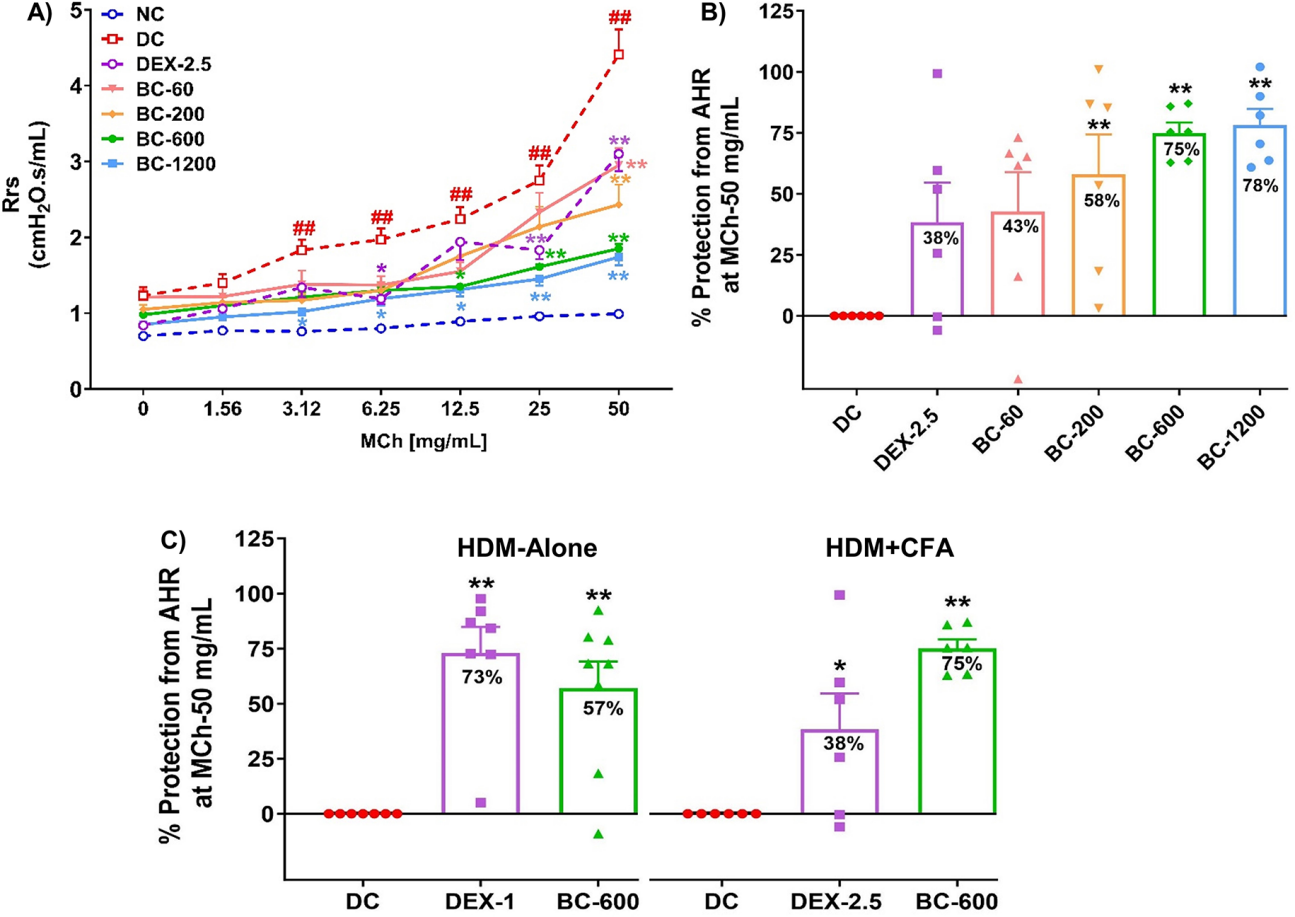
Bronchom pre-treatment prevents the development of airway hyperresponsiveness (AHR) in a dose-related manner. The animals were exposed to increasing concentrations of aerosolized Methacholine (MCh), twenty-four hours after the last HDM-instillation. (**A**) Depicts the bronchospasmogenic reaction to MCh at concentrations varying from 0 to 50 mg/mL. (**B**) Represents the percentage protection from AHR, computed by normalizing the respiratory system resistance (Rrs) values in mice assigned to groups 2 to group 7, which were obtained after challenging the mice with MCh-50 mg/mL, with the Rrs values observed in the normal-control group (group 1). Values within the bars represent percentage protection. (**C**) Represents the comparative analysis of the effects of dexamethasone and in murine models of allergic, eosinophil predominant asthma and mixed granulocytic asthma respectively. The data for the in-vivo effectiveness of dexamethasone-1 mg/kg in mouse model of HDM-alone-induced allergic asthma has been obtained from the previous study conducted at our laboratory (Balkrishna et al. 2024). Data is presented as mean ± S.E.M (*n* = 6 animals per group). For Fig. 3A, the data was analysed by two-way ANOVA followed by Tukey’s multiple comparison test, whereas for Fig. 3B and C, the data was analyzed by one-way ANOVA followed by Dunnett’s multi-comparison post hoc test. ##; *p* < 0.01 between normal-control group and disease-control group, *, *p* < 0.05; ** *p* < 0.01 between disease-control group and various treatment groups

When compared to saline-challenged (normal-control) animals, intranasal exposure of HDM for four consecutive days to the mice previously sensitized with HDM and CFA, resulted in a robust development of AHR to nebulized methacholine. Statistically significant differences between the normal-control (NC) and disease-control (DC) animals were evident from MCh-3.12 mg/mL to the highest concentration employed i.e. 50 mg/mL (Fig. 3A; *p* < 0.01). Oral administration of Bronchom (BC) at the doses of 60, 200, 600 and 1200 mg/kg, b.i.d. inhibited the development of AHR, when compared to the disease-control group (Fig. 3A; *p* < 0.05 at MCh-6.25 mg/mL for Bronchom-1200 mg/kg, b.i.d.; *p* < 0.05 at MCh-12.5 mg/mL for Bronchom-600 and 1200 mg/kg, b.i.d.; *p* < 0.01 at MCh-25 mg/mL for Bronchom-600 and 1200 mg/kg, b.i.d. and *p* < 0. 01 at MCh-50 mg/mL for all the tested doses of Bronchom). Dexamethasone (DEX) administered orally at a high dose of 2.5 mg/kg, also inhibited AHR (Fig. 3A; *p* < 0.05 at MCh-6.25 mg/mL and *p* < 0.01 at MCh-25 and 50 mg/mL). Additionally, in order to analyze the differences between the magnitude of protection from AHR between Bronchom and dexamethasone, we normalized the increase in Rrs noted in disease-control animal with that demonstrated by the normal-control animals at MCh-50 mg/mL and subsequently calculated the percentage protection from AHR for all the treated groups. By employing this analysis, Bronchom elicited a dose-related protection, which was statistically significant at the doses of 200, 600 and 1200 mg/kg, b.i.d., respectively (Fig. 3B; *p* < 0.01). Contrastingly, dexamethasone demonstrated only 38% protection from the development of AHR, which however was not statistically significant, when compared to the disease-control animals (Fig. 3B). Furthermore, we performed a comparative analysis of the percentage protection elicited by dexamethasone at the dose of 1 mg/kg/day, in mouse model of HDM-induced allergic asthma, previously conducted at our laboratory (Balkrishna et al. 2024), with the percentage protection obtained in the current study at 2.5-fold higher dose of dexamethasone. From this swift analysis it is clearly evident that dexamethasone-1 mg/kg/day, demonstrated a highly significant protection from the development of AHR (73%) when the animals were challenged alone with HDM. However, in the current study even a 2.5 times higher dose of dexamethasone could elicit only 38% protection, signifying markedly reduced steroid sensitivity in the current study model (Fig. 3C). In contrast, Bronchom-600 mg/kg, b.i.d. demonstrated a 57% protection from HDM-induced AHR and even better protection (75%) in HDM-CFA driven AHR at the same dose.

### Bronchom inhibits HDM-CFA driven leukocytosis, eosinophilia and neutrophilia in the BALF

**Fig. 4:**
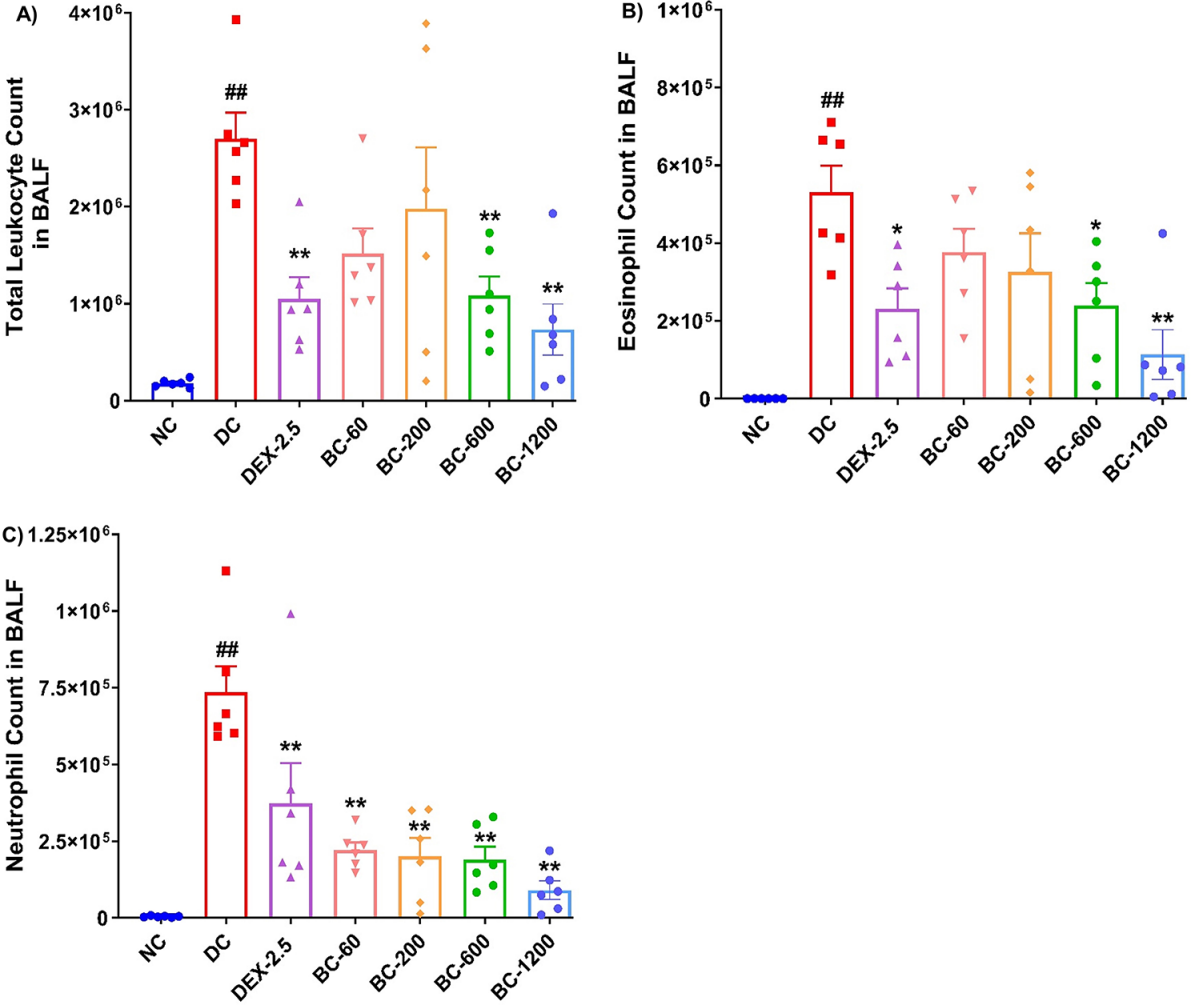
Bronchom pre-treatment decreases inflammatory cell influx in BALF. Animals were humanely sacrificed as detailed in the Materials and Methods section to compute (**A**) Total leukocyte counts (**B**) Eosinophil count and (**C**) Neutrophil count. Data is presented as Mean ± S.E.M (*n* = 6 animals per group). Data was analyzed by one-way ANOVA followed by Dunnett’s multi-comparison post hoc test. ##; *p* < 0.01 between normal-control group and disease-control group. *, *p* < 0.05; **, *p* < 0.01 between disease-control group and various treatment groups

HDM-challenge in HDM-CFA sensitized animals produced a statistically significant elevation in the total leukocytes and absolute eosinophil and neutrophil counts in the BALF, when compared to the normal-control animals (Fig. 4A-C; *p* < 0.01). The proportion of eosinophils and neutrophils in this model were comparable, indicating successful development of a mixed granulocytic asthma model. Bronchom, administered by oral route inhibited the observed leukocytosis in the BALF (Fig. 4A; *p* < 0.01 at Bronchom-600 and 1200 mg/kg, respectively. Dexamethasone administered orally at a high dose of 2.5 mg/kg reduced the increase in total leukocyte count in the BALF (Fig. 4A; *p* < 0.01), however it could not completely attenuate the HDM-CFA evoked leukocytosis. Bronchom also inhibited the influx of eosinophils in the BALF (Fig. 4B; *p* < 0.05 for Bronchom-600 mg/kg, b.i.d. and *p* < 0.01 for Bronchom-1200 mg/kg, b.i.d, respectively). Oral administration of dexamethasone at the dose of 2.5 mg/kg, also elicited a significant inhibition of HDM-induced eosinophilia (Fig. 4B, *p* < 0.01), but it did not completely abrogate the influx of eosinophils. Further, Bronchom also significantly inhibited the influx of neutrophils in the BALF (Fig. 4C; *p* < 0.01 for all the tested doses). Dexamethasone also demonstrated inhibition of HDM-CFA-induced neutrophilia (Fig. 4C, *p* < 0.01), which as in the case of leukocytosis and eosinophilic influx was not completely inhibited.

### HDM-CFA-driven influx of inflammatory cells in the lungs and goblet cell metaplasia is effectively curbed with Bronchom

**Fig. 5:**
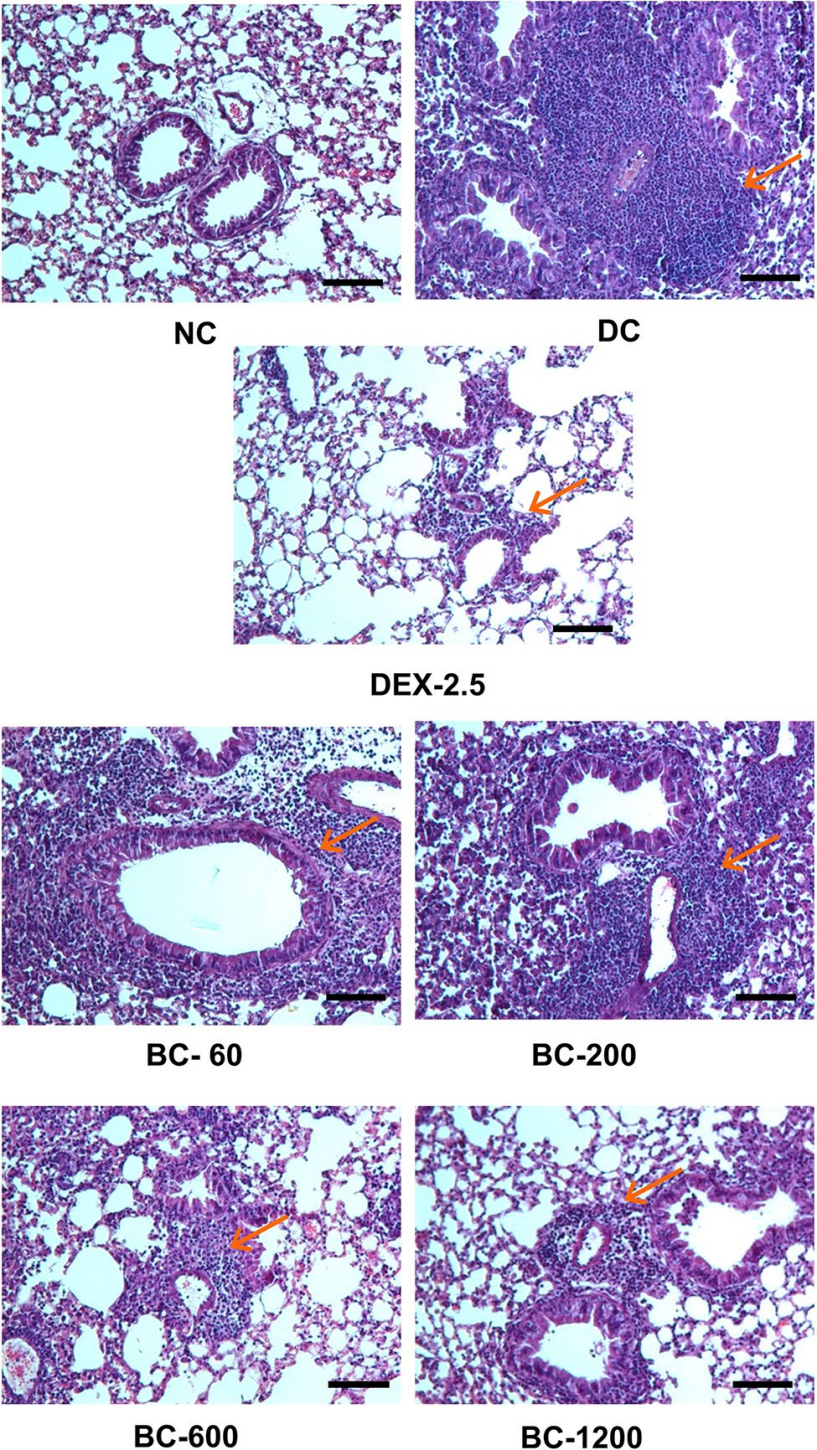
Bronchom reduces peribronchiolar and perivascular inflammation. Left lungs were harvested from animals and subjected to Haematoxylin and Eosin (H&E) staining. Representative sections of the lungs (× 100) are depicted from following study groups: Normal-control, (NC), Disease-control (DC), Dexamethasone (DEX: 2.5 mg/kg, q.d.) and Bronchom (BC: 20–600 mg/kg, b.i.d.), respectively. Red arrow indicates inflammatory cell infiltration. Scale Bar = 100 μm

**Fig. 6:**
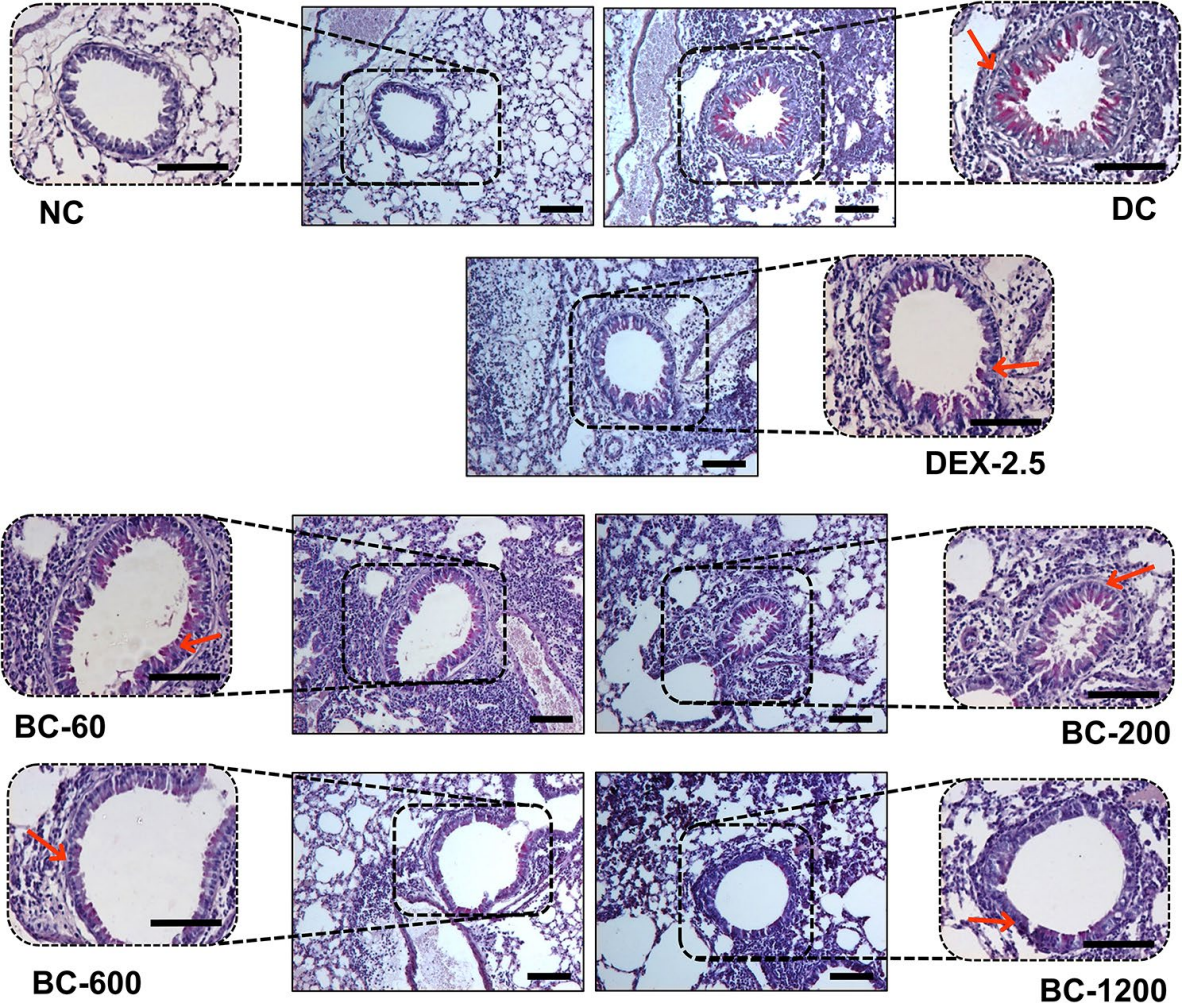
Bronchom decreases HDM-induced goblet cell metaplasia. Left lungs were excised from animals and processed for Periodic acid–Schiff staining (PAS). Representative photomicrographs of the lungs (× 100 and × 200) are depicted from the following experimental groups: Normal-control, (NC), Disease-control (DC), Dexamethasone (DEX: 2.5 mg/kg, q.d.) and Bronchom (BC: 20–600 mg/kg, b.i.d.), respectively. The photomicrographs where the dashed black boxes are portrayed have been obtained at 100 times magnification. Additionally, the area within the dashed boxes has been acquired at 200 times magnification and has been positioned next to the lower magnification photomicrograph. Higher magnified images have been displayed to show goblet cell metaplasia, which is depicted by the red arrow. Scale Bar = 100 μm

Repeated intranasal exposure of sensitized animals to HDM lead to peribronchiolar and perivascular inflammation, when compared to normal-control animals (Fig. 5). Additionally, goblet cell metaplasia was also clearly evident in disease-control animals (Fig. 6). Oral administration of Bronchom dose-dependently decreased the inflammatory cell infiltration in the peri-bronchial and peri-vascular areas. Furthermore, Bronchom was also able to reduce HDM-induced goblet cell metaplasia in a dose-related manner (Fig. 6). Dexamethasone also inhibited inflammation (Fig. 5) and goblet cell metaplasia (Fig. 6).

### Bronchom diminutes the HDM-CFA-evoked release of Th2 and pro-inflammatory cytokines in the BALF

**Fig. 7:**
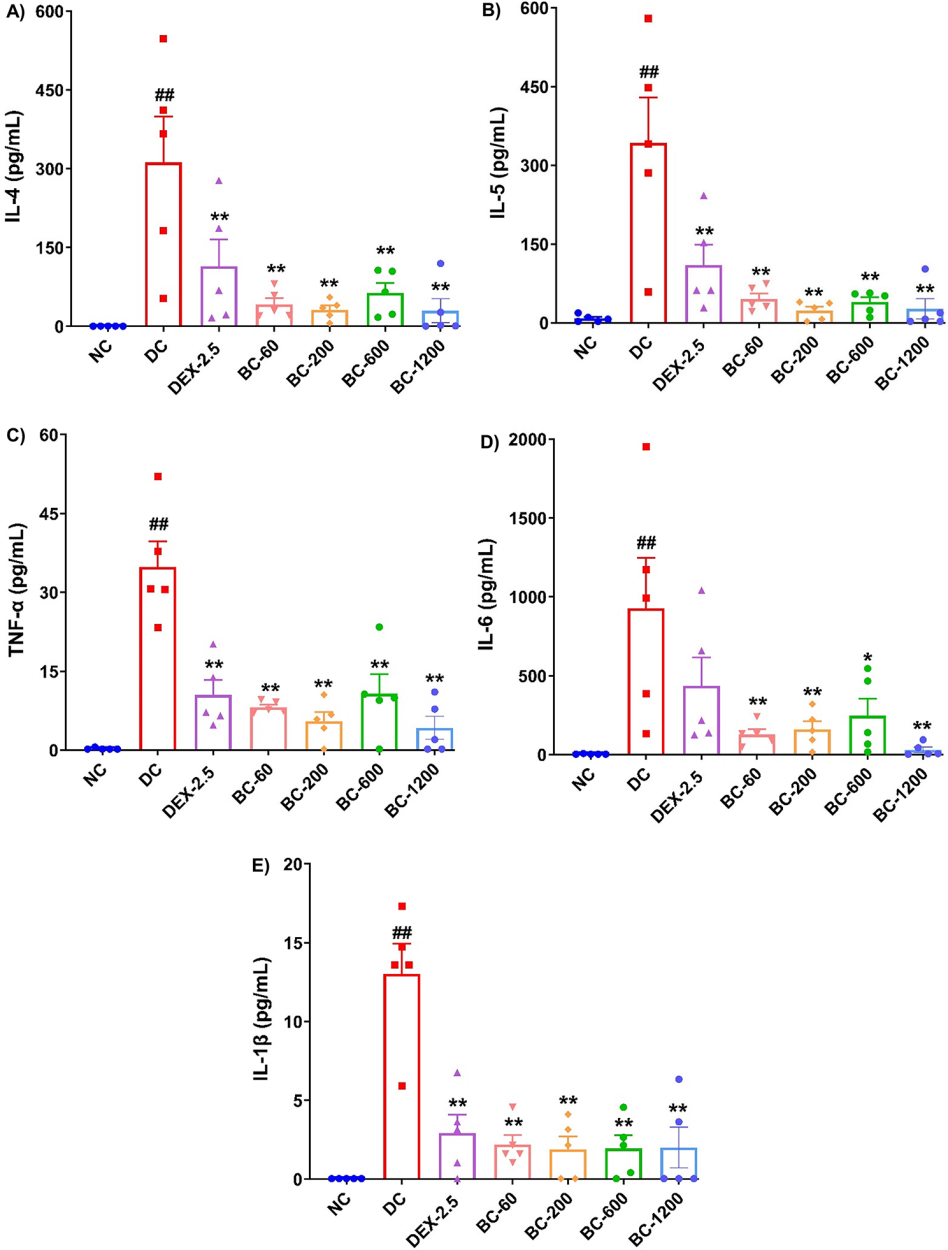
Bronchom reduces the levels Th2 and pro-inflammatory cytokines in BALF. Selected Th2 and pro-inflammatory cytokines were estimated in the BALF by multiplexing as mentioned in the in the Material and Methods section and expressed in pg/mL. **A**) IL-4, **B**) IL-5, **C**) TNF-α **D**) IL-6 and **E**) IL-1β. Data is presented as Mean ± S.E.M (*n* = 5 animals per group) and was analyzed by one-way ANOVA followed by Dunnett’s multi-comparison post hoc test. ##, *p* < 0.01 between normal-control group and disease-control group. *, *p* < 0.05, **, *p* < 0.01 between disease-control group and various treatment groups

HDM intranasal challenge in HDM-CFA sensitized animals provoked an enhanced secretion of Th2 cytokines: IL-4 and IL-5 as well as the pro-inflammatory cytokines, namely TNF-α, IL-6 and IL-β in the BALF when compared to the normal-control animals (Fig. 7A-E, *p* < 0.01). Bronchom demonstrated inhibition of HDM-induced increase in the levels of the evaluated cytokines. For IL-4 the effect was statistically significant at all the tested doses of Bronchom (Fig. 7A, *p* < 0.01), as compared to the disease-control group. A similar observation was also noted, for IL-5, wherein Bronchom significantly inhibited the HDM-induced increase in IL-5 levels in BALF (Fig. 7B, *p* < 0.01). Further, Bronchom effectively suppressed the elevated TNF-α levels in the BALF at all the evaluated doses (Fig. 7C, *p* < 0.01). Additionally, the HDM-provoked elevation of IL-6 was also effectively inhibited by Bronchom at all the tested doses (Fig. 7D, *p* < 0.05 at 600 mg/kg, b.i.d. and *p* < 0.01 at 60, 200 and 1200 mg/kg, b.i.d.), respectively. Finally, Bronchom also demonstrated significant efficacy in inhibiting the elevated levels of IL-1β at all the evaluated doses (Fig. 6E, *p* < 0.01). Reference standard dexamethasone was also able to significantly inhibit the elevated levels of IL-4 (Fig. 7A, *p* < 0.05), IL-5 (Fig. 7B, *p* < 0.05), TNF-α (Fig. 7C, *p* < 0.01) and IL-1β (Fig. 7E, *p* < 0.001), when compared to the disease-control group. However, unlike other cytokines, dexamethasone did not significatly inhibit the disease-induced elevated levels of IL-6, when compared to the disease-control group. (Fig. 7D).

### HDM-CFA driven mRNA expression of pro-inflammatory cytokines and MUC5AC is remarkably decreased with Bronchom

**Fig. 8:**
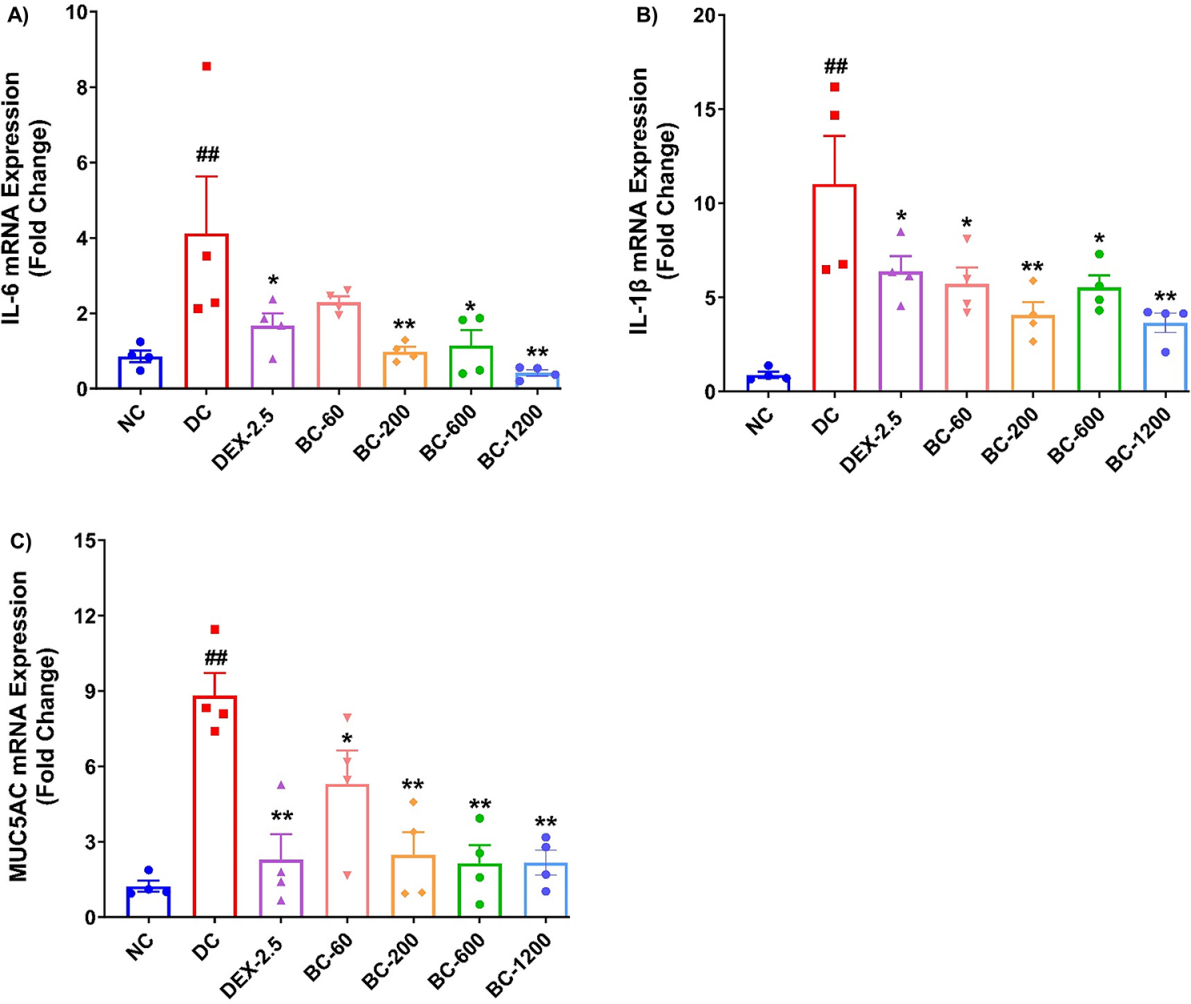
Bronchom attenuates the expression of pro-inflammatory cytokines and MUC5AC in the lungs. The mRNA expression of the selected cytokines and mucin in the lung tissue was assessed by qRT-PCR technique. (**A**) IL-6, (**B**) IL-1β and (**C**) MUC5AC. Data is presented as mean ± S.E.M (*n* = 4 animals per group) and was analyzed by one-way ANOVA followed by Dunnett’s multi-comparison post hoc test. ##, *p* < 0.01 between normal-control group and disease-control group. **, *p* < 0.01 between disease-control group and various treatment groups

The mRNA expression of the two evaluated pro-inflammatory cytokines associated with asthma development, namely IL-6 and IL-1β were significantly elevated in the lungs of HDM-challenged mice previously sensitized with HDM and CFA (Fig. 8A and B, *p* < 0.01), when compared to the normal-control animals. Similarly, the mRNA expression of the gene encoding for the predominant mucin, MUC5AC was also significantly increased (Fig. 8C, *p* < 0.01). Bronchom reduced the mRNA expression of all the three evaluated molecular markers when compared to the disease-control group. For IL-6, the effect was significant at 200, 600 and 1200 mg/kg, b.i.d. (Fig. 8A, *p* < 0.01). In case of IL-1β, the effect of Bronchom was significant at all the tested doses (Fig. 8B, *p* < 0.05 and 60 and 600 mg/kg, b.i.d. and *p* < 0.01 at 200 and 1200 mg/kg, b.i.d.). Similarly, for MUC5AC, the effect was significantly different from the disease-control group (Fig. 8A-C, *p* < 0.05 at 60 mg/kg, b.i.d. and *p* < 0.01 at 200, 600 and 1200 mg/kg, b.i.d.). Dexamethasone also significantly reduced the mRNA expression of the assessed markers in the lung, as compared to the disease-control group (Fig. 8A-C, *p* < 0.05 for IL-6 and IL-1β and *p* < 0.01 for MUC5AC).

## Discussion

Severe asthma is associated with high dose corticosteroid dependence, wherein a patient demonstrates worsening of asthma symptoms after reducing the dose of steroid or its discontinuation. In addition, steroid refractoriness or complete insensitivity to steroids may also be observed in severe asthmatics (Chung et al. 2014). A patient with severe asthma may also present with mixed sputum granulocytosis wherein, both eosinophilia and neutrophilia are evident and this particular phenotype is considered to be one of the most severe forms (Hastie et al. 2020). Continued use of corticosteroids, especially oral or systemic steroid therapy, for achieving asthma control is not a favorable proposition from a risk *vs.* benefit perspective, due to the steroid-associated acute and chronic complications. Acute complications may include a propensity to develop frequent infections ultimately leading to pneumonia, in most circumstances, as well as gastrointestinal complications like ulcers/bleeding and gastroesophageal reflux. Chronic administration on the other hand may adversely impact metabolism, skeletal system, cardiovascular system, nervous system and the eye. Consequently, the patients are at the risk of developing co-morbidities such as diabetes, obesity, osteoporosis, hypertension, hypercholesterolemia, anxiety, depression, sleep disorders and cataracts. Adding to the obvious impact on the general health of a severe asthmatic, long-term systemic steroid therapy is also associated with enhanced healthcare costs (Bleecker et al. 2020). Consequently, severe asthmatics often require add-on maintenance biological therapies to achieve optimal control and several such agents are available which target IgE, Th2 cytokines, IL-25 and IL-33 (Pelaia et al. 2020). However, as stated earlier the affordability and accessibility to such patient-tailored therapies, particularly in third world countries is rather challenging. Therefore, novel, efficacious, safe, affordable treatments that additionally ensure an excellent patient compliance are required, which can act as adjunct therapies, along with the conventional treatments to achieve the optimal control of symptoms in severe asthmatics. In this light, use of complementary and alternative medicines, derived from plants and minerals, is a promising approach (Balkrishna et al. 2020).

Bronchom, an Ayurvedic herbo-mineral prescription medicine, which is particularly indicated for the treatment of obstructive as well as interstitial lung diseases. Previously, we have reported that Bronchom demonstrates robust pharmacological effects in murine model of atopic asthma induced by HDM. (Balkrishna et al. 2024). In the present study, the preclinical in-vivo efficacy of Bronchom was assessed in mouse model of mixed granulocytic asthma induced by HDM extract and CFA. We opted to evaluate the in-vivo effectiveness of Bronchom in female mice, on the basis of a previous published pioneering study, which reported the development of HDM and CFA-induced mixed granulocytic asthma. In addition, the outcomes of the study clearly demonstrated absence of any sex-related differences in the responses of animals in the model (Menson et al. 2020). Murine models of mixed granulocytic asthma have been reported to develop after administration of ovalbumin and CFA (Ponci et al. 2020), cockroach extract (Nadeem et al. 2023) and intranasal challenge of ovalbumin and lipopolysaccharide in mice previously sensitized to ovalbumin (Xiao et al. 2022). We selected HDM to evoke airway inflammation as it is a clinically relevant, perennial antigen, and has been reported to be the most common disease-causative agent (Virchow et al. 2016). HDM is known to trigger the release of allergic asthma related cytokines, namely IL-4, IL-5 and IL-13 from Th2 cells, innate immune cells and innate lymphoid cells thereby driving eosinophil-predominant airway inflammation (Gao et al. 2022). Administration of CFA on the other hand results in Th1 (non-Th2) responses, leading to elevations of Th1 cytokines like TNF-α as well as pro-inflammatory cytokines like IL-6 and IL-1β and consequently causing the influx of neutrophils in the airways with reduced steroid sensitivity (Menson et al. 2020). The outcomes of the current study demonstrate that mixed granulocytic airway inflammation, successfully developed in the mice model along with AHR to aerosolized methacholine, and relevant histopathological, biochemical and molecular alterations. Additionally, steroid sensitivity was decreased as evidenced by a reduced efficacy of a high dose of dexamethasone in preventing the development of AHR. Moreover, although dexamethasone was able to reduce the infiltration of total leukocytes, eosinophils and neutrophils in the BALF, complete abrogation was not evident despite employing a high dose of the steroid in the study (2.5 mg/kg/d, as compared to 1 mg/kg/d, from our previously reported study for HDM-induced allergic asthma). In contrast, Bronchom demonstrated a robust pharmacological response by preventing the development of HDM-CFA induced functional, cellular, histological, biochemical and molecular changes.

Airway hyperresponsiveness to non-specific bronchospasmogenic agents is a cardinal feature of asthma, which is thought to be a consequence of airway remodeling. In the current study, Bronchom pre-treatment dose-dependently protected the mice from AHR. Notably, at the highest concentration of methacholine used in the study, significant protection was evident at all the tested doses of Bronchom. Interestingly, as mentioned earlier dexamethasone exhibited a diminished pharmacological response, clearly demonstrating that Bronchom possesses preclinical potential to inhibit AHR in a murine model of reduced steroid sensitivity.

The association of high sputum eosinophilia with the development of severe asthma and subsequent exacerbations has been well established, especially in the Th2 high endotype (Matucci et al. 2019). Consequently, an agent, which effectively suppresses eosinophilia could be a potential addition to the available therapeutic agents for severe asthma treatment. In the present study, Bronchom dose-dependently reduced antigen-induced eosinophilia suggesting that the Ayurvedic medicine has potential to address elevated eosinophils observed in severe asthmatics. In mouse model of allergic eosinophilic predominant asthma induced by HDM alone, dexamethasone almost completely inhibits the influx of this particular granulocyte (Balkrishna et al. 2024) at a low dose of 1 mg/kg/day. However, in the current study, it did not completely abolish eosinophilia even at a high dose, which may in part explain the observed reduced effectiveness of dexamethasone in protecting the mice from the development of AHR.

In mixed granulocytic asthma, influx of neutrophils occurs along with eosinophils in the airways, particularly due to an involvement of the Th1 cytokine axis. Appearance of neutrophils in the sputum of asthmatics directly correlate with disease severity, asthma exacerbations and steroid refractoriness. Moreover, there are no effective treatments available to attenuate neutrophilia (Panettieri 2018). In the present study we were able to note infiltration of neutrophils in the BALF, as well. More importantly, the population of both the granulocytes were comparable, with neutrophils being numerically higher as compared to eosinophils. High dose of dexamethasone did decrease BALF neutrophila but Bronchom exhibited a greater efficacy. Accordingly, the encouraging results of the present study suggest that Bronchom also possesses the potential to reduce neutrophil influx observed in severe asthmatics with a mixed granulocytic phenotype, for which, no therapeutic modality currently exists.

Histopathological analysis of lung tissue of disease control animals revealed severe infiltration of inflammatory cells as well as goblet cell metaplasia, which was inhibited by Bronchom dose-dependently. This suggests that the prescription medicine has the potential to ameliorate the airway remodeling changes and the resultant AHR.

Further, we also assessed the concentrations of Th2 cytokines in the BALF, namely, IL-4 and IL-5 in the study. Both of these cytokines were found to be elevated in the BALF of disease control mice. IL-4 is involved in eosinophil activation, synthesis of immunoglobulin E, mucus secretion and remodeling of the airways. Based on this premise, Dupilumab, an IL-4 receptor antagonist, is indicated for the treatment of severe asthma (Bagnasco et al. 2016). The role of IL-5 in maturation, development, and actions of the eosinophil is well known and monoclonal antibodies directed towards IL-5 (mepolizumab) and its receptor (benralizumab) are available in the clinics as an adjunct therapy for severe hypereosinophilic asthma (Bagnasco et al. 2018). In the present study, Bronchom potently decreased the levels of both IL-4 and IL-5, which may partly explain its effectiveness in lowered eosinophil counts, airway remodeling changes and AHR.

In addition to Th2 cytokines the effect of Bronchom was also evaluated on the BALF concentrations of pro-inflammatory cytokines: TNF-α, IL-6 and IL-1β and the mRNA expression of IL-6 and IL-1β in the lung. In the BALF, elevations in the levels of all the three cytokines was observed, which was potently suppressed by Bronchom pre-treatment. Moreover, the magnitude of inhibition demonstrated by Bronchom was greater than the high dose of dexamethasone. Further, in molecular studies, Bronchom decreased the mRNA expression of IL-6 and IL-1β, The studied cytokines have been implicated in the pathogenesis of severe mixed granulocytic asthma, with a predominant neutrophil component and consequently exhibiting reduced steroid sensitivity (Kim et al. 2017; Menson et al. 2020; Niessen et al. 2021). Accordingly, the results of biochemical and molecular studies may explain in part, the observed decrease in BALF neutrophils.

Bronchial mucus plugs are frequently noted in patients with severe asthma, which ultimately contribute to AHR (Dunican et al. 2018). Accordingly, we also assessed the mRNA expression of MUC5AC in lungs of the mice. MUC5AC is one of the predominant gel forming mucin, whose mRNA expression has been reported to be elevated in patients with severe neutrophilic asthma (Khorasani et al. 2023). In the present study, Bronchom effectively decreased the expression of MUC5AC mRNA, which is again an encouraging finding as coupled with a reduction in goblet cell metaplasia, the results provide preclinical evidence of the effectiveness of Bronchom in reducing mucus secretion.

Finally, the HPTLC and UHPLC analysis of Bronchom divulged the presence of bioactive marker compounds, which have been reported to possess anti-oxidant, anti-inflammatory and anti-allergic activities. Accordingly, these phytometabolites possess potential to modulate the airway disorder-related pathophysiology of the lungs. Of these compounds, gallic acid, a known effective anti-oxidant has been reported to moderate ovalbumin-induced allergic airway inflammation in mice by inhibiting the release of Th2 cytokines (Wang et al. 2018). Additionally, gallic acid has also demonstrated protection from acute lung injury in rats induced by lipopolysaccharide (LPS), by reducing the blood levels of TNF-α, which is a Th1 cytokine as well as IL-1β and IL-6, which are the other pro-inflammatory cytokines (Kardaş et al. 2024). Similarly, glycyrrhizin another phytometabolite detected in Bronchom has demonstrated alleviation of ovalbumin-evoked allergic asthma in a murine model by inhibiting all the cardinal features associated with the disease, including the blunting of Th2 response (Ram et al. 2006). Likewise, it also demonstrated attenuation of LPS-induced acute lung injury in mice by lowering the levels of TNF-α, IL-1β and IL-6 (Wang et al. 2023). Further, in mouse model of ovalbumin-induced experimental asthma, piperine, another phytocompound present in Bronchom has demonstrated preclinical effectiveness in ameliorating the disease-associated functional and cytological alterations by lowering Th2 cytokines and chemokines (Kim and Lee 2009). Similar to gallic acid and glycyrrhizin, piperine has also demonstrated protection from LPS-induced acute lung injury in mice, primarily characterized by airway neutrophilia, by attenuating the levels of TNF-α, IL-1β and IL-6 (Lu et al. 2016). In addition, piperine has also been reported to lower cigarette smoke-induced pulmonary neutrophilic inflammation, a Th1-driven model by blunting the release of TNF-α, IL-1β and IL-6 (Saha et al. 2022). Eugenol, also detected in Bronchom has also been evaluated for its in-vivo efficacy in a murine model of atopic asthma induced by ovalbumin, wherein it suppressed AHR, eosinophilic influx in BALF and the lungs as well as the release of IL-4 and IL-5 (Pan and Dong 2015). Additionally, eugenol improved LPS-induced acute lung injury in mice by diminution of TNF-α, IL-1β and IL-6 (Magalhães et al. 2019). Furthermore, the anti-asthmatic effects of methyl gallate, another phytometabolite present in Bronchom has been evaluated in guinea pigs sensitized with ovalbumin, wherein it reduced the allergen as well as platelet activating factor-1 evoked bronchial hyperreactivity (Dorsch et al. 1992). Similarly, rosmarinic acid, another phytocompound revealed by phytochemical analysis of Bronchom has demonstrated anti-asthmatic activity in ovalbumin-induced experimental asthma in mice (Liang et al. 2016). Additionally, rosmarinic acid alleviated LPS-induced acute respiratory distress syndrome in mice, wherein it lowered the expression of TNF-α and IL-1β in the lung at protein level (Zeng et al. 2024). Protocatechuic acid, a known anti-inflammatory agent detected in Bronchom has been reported to suppress ovalbumin-induced allergic airway inflammation and the resultant aberrations in lung function by reducing Th2 cytokines and mucin expression in mice (Wei et al. 2013). In addition, protocatechuic acid demonstrated protection against the development of LPS-induced acute lung injury in mice by lowering the levels of TNF-α and IL-1β (Zhang et al. 2015). Further, 6-gingerol has demonstrated pharmacological effectiveness in mouse model of ovalbumin-induced experimental atopic asthma as evidenced by decreased lung inflammation and mucus secretion, presumably due to its anti-oxidant activity (Kim et al. 2021). Additionally, 6-gingerol suppressed LPS-induced acute lung injury in rats by reducing the lung contents of TNF-α, IL-1β and IL-6 (Pan et al. 2023). Taken together, these literature evidences suggest that the majority of phytometabolites present in Bronchom demonstrate activities both in eosinophilic asthma as well as neutrophilia-driven acute lung injury. In the current study we have observed decrease in both Th2, Th1 and pro-inflammatory cytokines. As a corollary, it can be presumed that the phytometabolites present in Bronchom might have contributed to its observed preclinical effectiveness in mixed granulocytic model reported in the current study. However, it is challenging to ascertain as to which phytocompound may have majorly contributed to the observed in-vivo efficacy of Bronchom. Nonetheless, extraction of the published information on various phytocompounds of Bronchom, would reveal that pharmacological effects of eugenol (Pan and Dong 2015), rosmarinic acid (Liang et al. 2016), piperine (Lu et al. 2016) and gallic acid (Kardaş et al. 2024) would occur at the doses of 10–20 mg/kg, 20 mg/kg, 30–60 mg/kg and 100 mg/kg, respectively. On the basis of HPTLC analysis, the relative doses of these phytocompounds in Bronchom at the doses of 60, 200, 600 and 1200 mg/kg range from 0.03 mg/kg to 5.12 mg/kg. Given this quantitative assessment of sub-optimal doses of each phytocompound, and observed pharmacological effects of Bronchom, it is conceivable that that these phytocompounds may work in a synergistic fashion and could consequently improve asthma symptoms.

There is limited literature in the public domain about the preclinical in-vivo effectiveness of herbal or herbo-mineral medicines in mixed granulocytic asthma. It has been reported that sensitization and subsequent challenge of mice to ragweed extract leads to development of a neutrophilic predominant asthma, which exhibits steroid resistance and a herb-based formulation, ASHMI demonstrates in-vivo efficacy in reducing lung inflammation (Srivastava et al. 2014). However, to the best of our knowledge this is the first report of the pharmacological effectiveness of a herbo-mineral medicine in an animal model of HDM and CFA-induced mixed granulocytic airway inflammation. In public domain there are no published studies, which have evaluated the effects of herb-based formulations or a small molecule in the murine model reported in the present study, to contrast with, underscoring the necessity for additional research in coming times.

With an objective of demonstrating proof of concept, we administered Bronchom by following a prophylactic regimen, wherein the medicine was administered two-weeks prior to sensitization with HDM and CFA. Since we obtained positive results in the present study, future studies are needed to assess the in-vivo efficacy of Bronchom administered concurrently with HDM challenge in animals previously sensitized with HDM and CFA. Such a study would further substantiate the preclinical effectiveness of Bronchom. In addition, this study also opens the way to assess the effect of Bronchom in patients of mixed granulocytic asthma under clinical conditions. Furthermore, the observed effects of Bronchom in reducing neutrophil influx makes it as an effective candidate in testing its pharmacological effects in animal models chronic obstructive pulmonary disease.

## Conclusions

The current study has demonstrated the preclinical efficacy of a herbo-mineral prescription medicine, Bronchom in inhibiting the development of the characterisitic features of mixed granulocytic severe asthma, induced by combined administration of house dust mite and Complete Freund’s Adjuvant. The Ayurvedic medicine prevented the development of AHR, infiltration of total leukocytes, eosinophils and neutrophils in the BALF as well as in the lung. It also ameliorated HDM-CFA evoked goblet cell metaplasia. In addition Bronchom decreased the levels of Th2 and pro-inflammatory cytokines in the BALF and also reduced the mRNA expression of pro-inflammatory cytokines and MUC5AC in the lung. Nevertheless, the high dose of dexamethasone employed in the current study exhibited reduced efficacy in the development of AHR and did not completely abrogate the cytological, biochemical and molecular changes induced by HDM instillation in HDM and CFA-sensitized mice. Taken together, the findings of the study strongly suggest that Bronchom is a candidate for further clinical investigations as an adjunct therapy for the management of the symptoms of mixed granulocytic asthma in patients that exhibit reduced sensitivity to inhaled or systemic corticosteroid therapy.

## Data Availability

No datasets were generated or analysed during the current study.

## Abbreviations

AHR: Airway hyperresponsiveness
ANOVA: Analysis of variance
b.i.d.: *bis in die*
BALF: Bronchoalveolar lavage fluid
BC: Bronchom
CCSEA: Committee for Control and Supervision of Experiments on Animals
CFA: Complete Freund’s adjuvant
DEX: Dexamethasone
DC: Disease control
EU: Eugenol
GA: Gallic acid
GY: Glycyrrhizin
H&E: Hematoxylin and Eosin
HDM: House dust mite
HPTLC: High-performance thin layer chromatography
LPS: Lipopolysaccharide
MC: Methylcellulose
MCh: Methacholine
MG: Methyl gallate
MUC5AC: Mucin 5AC
NC: Normal control
p.o.: *per os*
PAS: Periodic acid-Schiff
PI: Piperine
PPIA: Peptidylprolyl Isomerase A
q.d.: *quaque die*
qRT-PCR: Quantitative Real-time Polymerase Chain Reaction
RA: Rosmarinic acid
Rrs: Respiratory system resistance
SEM: Standard error of mean
TLC: Thin layer chromatography
UHPLC: Ultra-high performance liquid chromatography
